# A single β-octyl glucoside molecule induces HIV-1 Nef dimer formation in the absence of partner protein binding

**DOI:** 10.1371/journal.pone.0192512

**Published:** 2018-02-07

**Authors:** Mousheng Wu, John J. Alvarado, Corinne E. Augelli-Szafran, Roger G. Ptak, Thomas E. Smithgall

**Affiliations:** 1 Department of Chemistry, Drug Discovery Division, Southern Research Institute, Birmingham, AL, United States of America; 2 Department of Microbiology and Molecular Genetics, University of Pittsburgh School of Medicine, Pittsburgh, PA, United States of America; 3 Department of Infectious Disease Research, Southern Research Institute, Frederick, MD, United States of America; University of Washington, UNITED STATES

## Abstract

The HIV-1 Nef accessory protein is essential for viral pathogenicity and AIDS progression. Nef forms complexes with multiple host cell factors to facilitate viral replication and promote immune escape of HIV-infected cells. Previous X-ray crystal structures demonstrate that Nef forms homodimers, the orientation of which are influenced by host cell binding partners. In cell-based fluorescence complementation assays, Nef forms homodimers at the plasma membrane. However, recombinant Nef proteins often exist as monomers in solution, suggesting that membrane interaction may also trigger monomer to dimer transitions. In this study, we show that monomeric Nef core proteins can be induced to form dimers in the presence of low concentrations of the non-ionic surfactant, β-octyl glucoside (βOG). X-ray crystallography revealed that a single βOG molecule is present in the Nef dimer, with the 8-carbon acyl chain of the ligand binding to a hydrophobic pocket formed by the dimer interface. This Nef-βOG dimer interface involves helix αB, as observed in previous dimer structures, as well as a helix formed by N-terminal residues 54–66. Nef dimer formation is stabilized in solution by the addition of βOG, providing biochemical validation for the crystal structure. These observations together suggest that the interaction with host cell lipid mediators or other hydrophobic ligands may play a role in Nef dimerization, which has been previously linked to multiple Nef functions including host cell protein kinase activation, CD4 downregulation, and enhancement of HIV-1 replication.

## Introduction

The *nef* genes of human and simian immunodeficiency viruses (HIV-1, HIV-2, and SIV) encode accessory proteins of 27–35 kDa critical for viral pathogenesis [1,2]. While Nef is not essential for HIV-1 replication *in vitro*, it enhances virus replication *in vivo* and promotes AIDS progression. Deletion of Nef attenuates SIV replication, pathogenicity and AIDS progression in non-human primates [3]. More recent studies have shown that Nef is also required for HIV-1 replication and CD4^+^ T cell loss in humanized mice [4,5]. Conversely, targeted expression of Nef alone to the CD4^+^ cellular compartment is sufficient to induce an AIDS-like syndrome in transgenic mice [6]. Consistent with these observations, some individuals infected with Nef-defective HIV-1 remain asymptomatic for many years in the absence of antiretroviral therapy [7,8]. These studies demonstrate the importance of Nef to HIV-1 pathogenesis *in vivo*, and support the development of drugs targeting this virulence factor as a new approach to HIV-1 therapy [9].

At the molecular level, Nef enhances viral infectivity and replication by stimulating multiple host cell signal transduction pathways. Important Nef partner proteins include members of the Src and Tec protein-tyrosine kinase families expressed in HIV-1 target cells, especially Hck and Itk [10–14]. Selective inhibitors of these Nef-activated kinase pathways block HIV-1 replication *in vitr*o [11,13,14]. Nef also hijacks endocytic trafficking pathways to induce downregulation of viral receptors, including CD4 and chemokine receptors, and prevent cell-surface display of MHC-I in complex with HIV-1 antigens [15]. These mechanisms allow HIV-infected cells to avoid immune surveillance via cytotoxic T lymphocytes (CTLs) [16] as well as antibody-dependent cell-mediated cytotoxicity [17]. Small molecule Nef inhibitors have recently been shown to restore cell-surface MHC-I to patient-derived CD4^+^ T cells *in vitro*, and trigger autologous CTL responses [18,19]. These findings illustrate the potential of Nef inhibitors to help the immune system eradicate HIV-infected cells [9].

Structural analyses of Nef and Nef-effector complexes by both NMR spectroscopy and X-ray crystallography have identified features critical for its function [20–26]. These features include an N-terminal flexible region (amino acids 1–50 in the HIV-1 SF2 isolate used in the present study; all numbering is based on the crystal structure of Nef NL4-3, PDB: 1EFN [22]), an acidic cluster (EEEE) and PXXP motif (62–77), a well-ordered core domain (79–206), and a central loop within the core domain (148–180). The N-terminal flexible region of Nef is myristoylated, which is essential for membrane association and function [27]. The acidic cluster interacts with the μ1 subunit of the clathrin adaptor protein complex 1 (AP-1), which in turn binds to the cytoplasmic tail of MHC-I to prevent cell surface display of viral antigens [26]. The PXXP motif forms a polyproline type II helix responsible for recruitment of protein kinases with SH3 domains, including the Src-family kinase Hck [22,23]. The folded core domain exhibits the highest degree of sequence and structural conservation among Nef proteins. The core consists of two anti-parallel α-helices, four anti-parallel β-strands, and two short α-helices at the C-terminus [22]. The central loop contains two short α-helices plus dileucine and diacidic motifs but is not observed in most Nef crystal structures due to its flexibility. However, a recent crystal structure of Nef in complex with the α and σ2 subunits of AP-2, the endocytic adaptor protein involved in CD4 downregulation, revealed the structure of the flexible loop. This loop makes multiple contacts with AP-2 essential for this Nef function [25].

X-ray crystallography has also revealed Nef homodimers, the formation of which appears to depend on the partner protein that is bound to Nef. For example, structures of Nef bound to Src-family kinase SH3 and SH3-SH2 domains revealed a dimer of Nef complexes, in which conserved hydrophobic residues present on the Nef helix αB form the dimer interface [22,23,28]. In contrast, Nef is present as a monomer in crystal complexes with subunits of the endocytic adaptors AP-1 and AP-2 as described above [25,26]. Cell-based fluorescence complementation studies have revealed that Nef alone forms dimers that localize to the cellular membrane [29]. Mutations in the Nef dimerization interface, based on the crystal structure of Nef bound to a Src-family kinase SH3 domain, disturb dimerization in the fluorescence complementation assay, suggesting that the crystal interface is relevant to Nef dimer formation at the membrane in cells. Importantly, mutations that impact Nef dimerization also dramatically reduce both Nef-induced CD4 downregulation and HIV-1 replication in cell culture [29]. More recently, dimerization was also shown to be important for Nef interaction with AP-2 using a similar cell-based fluorescence complementation assay [30]. Together, these observations suggest that Nef may exist in a dynamic equilibrium between monomer and dimer states, which are critical to its ability to interact with a diverse array of partner proteins.

Here we report a novel X-ray crystal structure of the HIV-1 Nef core region in which a dimer is observed in the absence of a partner protein. The structure revealed that the 8-carbon hydrophobic tail of a single molecule of β-octyl glucoside (βOG), a surfactant commonly used in recombinant protein expression and purification, bound to a deep pocket formed by the two αB helices that come together to form the Nef dimerization interface. In addition, we observed an α-helix near the Nef N-terminus (residues 54–66), which also contributes to the Nef dimer interface. Size-exclusion chromatography demonstrated that βOG also induced Nef dimerization in solution in the absence of other binding partners. Interaction of Nef with host cell lipid mediators, including the myristate modifications present on several Nef binding partners as well as Nef itself, may influence dimer formation in cells.

## Materials and methods

### Expression construct design and cloning

The pGEX-4T-3 vector (GE Healthcare Life Sciences) was engineered to replace the thrombin cleavage site (LVPRGS) with the tobacco etch virus (TEV) protease cleavage site (ENLYFQGS) to create the modified vector, pGEX-TEV. A cDNA fragment of the HIV-1 Nef (SF2 isolate) core domain (residues 51–205; numbering is based on the crystal structure of HIV-1 NL4-3 Nef PDB: 1EFN [22]) was amplified by PCR using the forward primer CGCGGATCCACTAATGCTGATTGTGCCTGGCTAGAAG and the reverse primer CGCGTCGACTTAGTCTTTGTAGTACTCCGGATGCAGC (*Bam*HI and *Sal*I restriction sites are underlined). The resulting PCR product was purified, digested with *Bam*HI and *Sal*I, and ligated into pGEX-TEV to create the final expression construct, pGEX-TEV-Nef. The Nef coding region was confirmed by DNA sequencing.

### Protein expression and purification

*E*.*coli* strain Rosetta (DE3) pLysS (EMD Millipore) was transformed with pGEX-TEV-Nef, and a single colony was picked and cultured overnight in LB medium at 37 °C. The overnight culture (10 mL) was used to inoculate 500 mL of auto-induction medium [31]. The cells were grown at 37 °C for 4 hours, followed by overnight induction at 18 °C. Cell pellets were resuspended in lysis buffer (20 mM Tris-HCl, pH 8.0, 500 mM NaCl, 2 mM DTT) supplemented with one SIGMAFAST protease inhibitor tablet and 1 mg/ml lysozyme (Sigma-Aldrich). Cells were disrupted using sonication, and the cell debris was removed by centrifugation at 16,000 rpm for 1 h using a Sorvall SS-34 rotor. The supernatant was loaded onto a Glutathione Sepharose 4B column (GE Healthcare Life Sciences) preincubated with lysis buffer and washed with 10 column volumes of buffer. The GST-Nef fusion protein was eluted in lysis buffer containing 25 mM reduced glutathione (Sigma-Aldrich). The eluted protein solution was mixed with GST-TEV protease (1:20, w/w; prepared in-house) and passed through a Sephadex G-25 desalting column (HiPrep 26/10, GE Healthcare Life Sciences) to remove the free glutathione. The protein mixture was incubated at 4 °C overnight and reloaded onto the Glutathione Sepharose 4B column to remove GST, GST-TEV and uncleaved GST-Nef protein. The cleaved Nef protein solution was collected and further purified by size-exclusion chromatography (SEC) using a Superdex 75 column (16/600, GE Healthcare Life Sciences) in SEC buffer (20 mM Tris-HCl, pH 8.0, 150 mM NaCl, 2 mM DTT). The fractions containing the Nef (51–205) protein were identified by SDS-PAGE, pooled and concentrated to 8 mg/ml in presence of 0.1% β-octyl glucoside (βOG) to prevent protein aggregation.

### Crystallization and structure determination

Purified Nef (51–205) was crystallized in 18–20% polyethylene glycol monomethyl ether 5000, 0.1 M Bis-Tris propane, pH 8.0, and 5% glycerol using the sitting-drop method. The crystals were cryo-protected in crystallization solution with 25% glycerol and flash-cooled in liquid nitrogen. Data were collected at Southeast Regional Collaborative Access Team (SER-CAT) 22-ID beamline (Advanced Photon Source, Argonne National Laboratory) and processed by XDS [32] and the CCP4 suite [33]. The structure of Nef was determined by molecular replacement using Phaser [34] and the structure of Nef from HIV-1 NL4-3 (PDB ID: 1AVV) [28] as the search model. The model was built in COOT [35] and refined by Refmac5 [36]. After several rounds of model building and refinement, the model was refined to R_work_ and R_free_ of 23.3% and 25.1% respectively. A simulated annealing omit map was generated using phenix.refine [37] to confirm the location of the βOG ligand and the novel N-terminal helical structure. The stereochemical quality of the final model was evaluated by PROCHECK [38]. All graphic representations of molecular structures were generated using PyMOL [39] (Schrӧdinger). The statistics of data processing and model refinement are summarized in Table 1. The final structure was deposited in the RCSB protein data bank (PDB ID: 6B72) and the coordinates will be released upon publication.

**Table 1:** Statistics of data collection, processing and refinement. Values in parentheses are for the outer shell. The free R factor was calculated using a randomly selected 5% of reflections omitted from the refinement.

**Data collection and process**	
Beamline	ID-22 (SER-CAT)
Wavelength (Å)	1.0
Temperature (K)	95
Detector	CCD, Rayonix(Mar) 300HS
Space group	P3221
a, b, c (Å)	109.58, 109.58, 247.04
α, β, γ (°)	90, 90, 120
Mosaicity (°)	0.12
Resolution range (Å)	94.9–3.2 (3.37–3.2)
Total unique reflections	29203 (4209)
I/sigI (I)	20.5 (3.2)
Completeness (%)	100 (100)
multiplicity	10.8 (10.7)
Rmerge	0.075 (0.828)
**Structure refinement**	
No. reflections, working set	27655
No. reflections, free set	1444
No. of non-hydrogen atoms	6243
Rwork/Rfree (%)	23.34/25.17
RMS deviations	
Bond distance (Å)	0.007
Bond angles (°)	1.167
Average B factor (Å ^2^)	86
Overall figure of merit (%)	78.79
Ramachandran plot	
Most favored regions (%)	89.1
Additional allowed (%)	10.9

### Analytical size-exclusion chromatography

Analytical size-exclusion chromatography (SEC) was performed on a Superdex 75 10/300 GL column (GE Healthcare Life Sciences) equilibrated with a solution of 20 mM Tris-HCl, pH 8.3, 150 mM NaCl, 10% (v/v) glycerol, 2 mM TCEP as the running buffer. All chromatography runs were conducted at a flow rate of 0.5 ml/min with sample volumes of 100 μl. SEC analysis of Nef (51–205) in the absence of βOG was conducted at a protein concentration of 10 mg/ml (549 μM). To determine the effect of βOG on Nef dimerization, Nef (51–205) (9.0 mg/ml; 494 μM) was incubated with different concentrations of βOG on ice for 15 min and then injected onto the SEC column equilibrated with running buffer containing the corresponding concentrations of βOG. Size-exclusion chromatography protein standards included bovine serum albumin (66 kDa), carbonic anhydrase (29 kDa) and cytochrome C (12.4 kDa) (Sigma-Aldrich).

## Results and discussion

### Overall structure of Nef (51–205)

**Fig 1:**
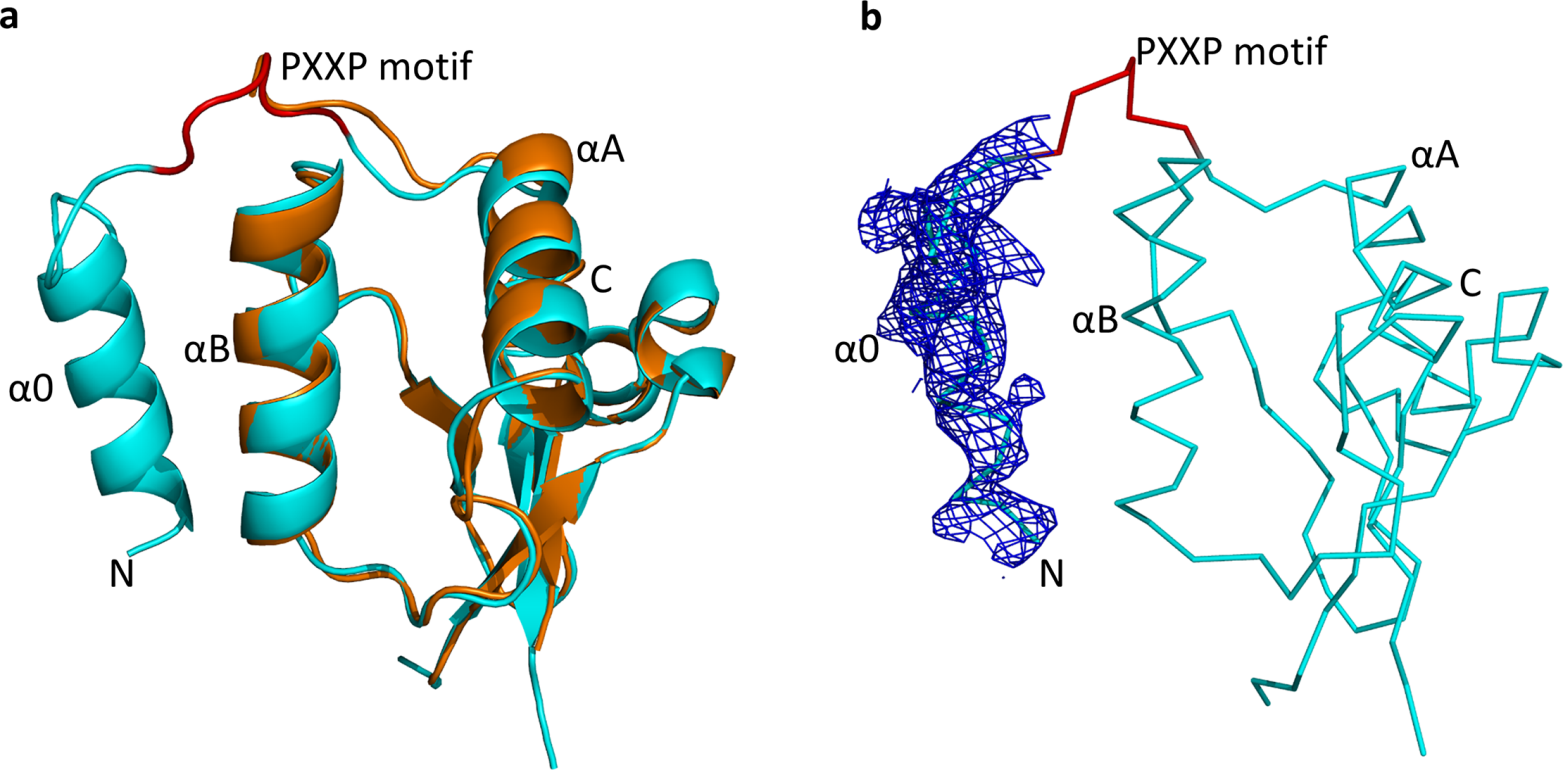
Overall structure of a Nef (51–205) monomer from the HIV-1 SF2 isolate. **(a)** The overall structure of Nef (51–205) (cyan ribbon) aligned with the structure of the Nef core from an SH3 domain complex (PDB ID: 1AVV; orange ribbon). **(b)** Simulated annealing omit map for helix α0. The backbone of the Nef (51–205) structure in shown as a ribbon with the PXXP motif labelled in red. The electron density map for helix α0 is rendered as blue mesh with a contour of 1.5 δ.

**Fig 2:**
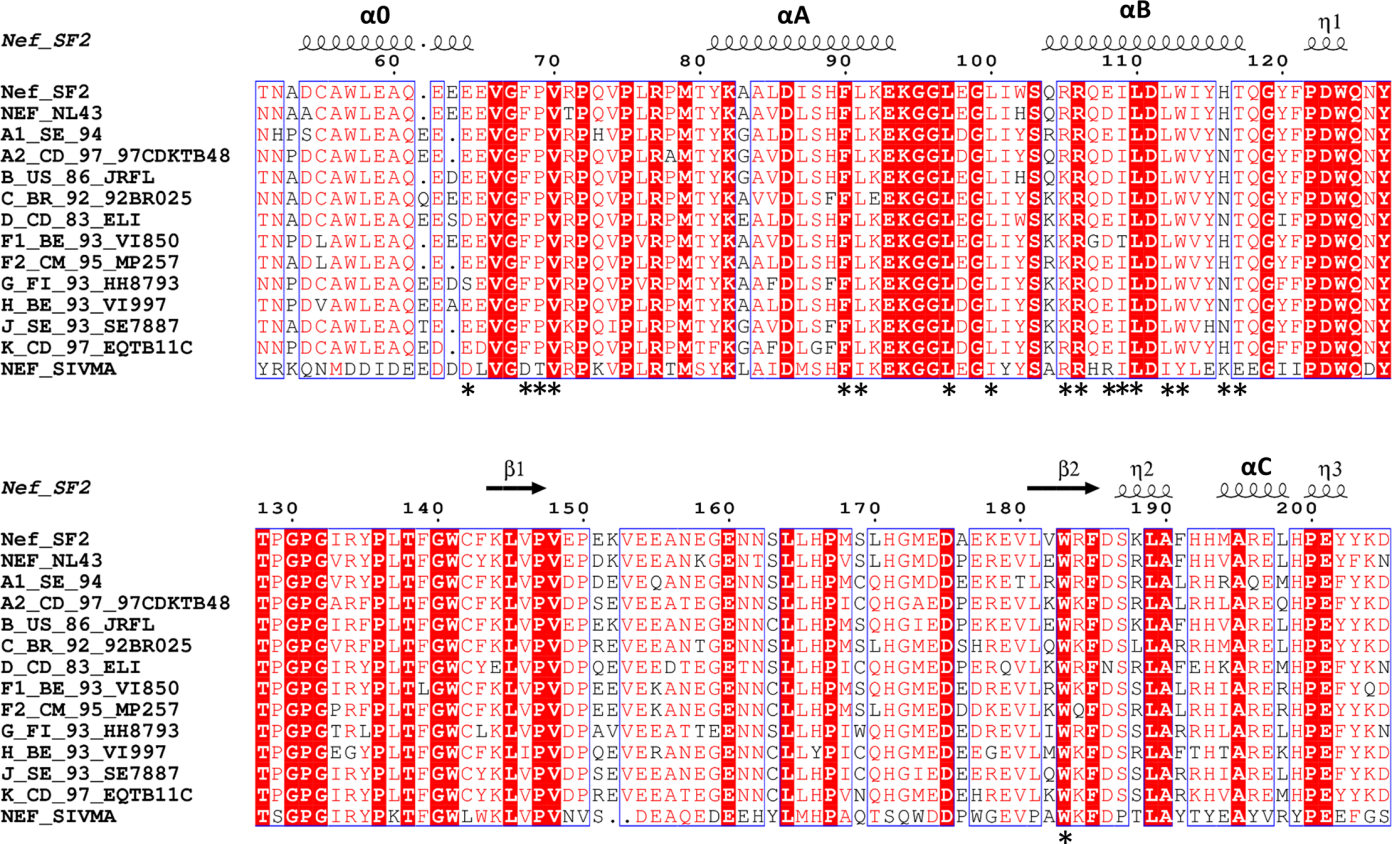
Sequence alignment of Nef proteins from representative strains of HIV-1 group M subtypes and SIV. Residue numbers above the sequences correspond to the HIV-1 Nef core structure from the B-clade HIV-1 isolate, NL4-3 (PDB: 1EFN [22]) The secondary structure elements of Nef (51–205) are indicated by arrows for strands and coils for helices. The residues involved in homodimer interaction in Nef (51–205) are labeled with asterisks (*) under the sequence alignment. Sequences shown include Nef_SF2: HIV-1 isolate SF2; NEF_NL43: HIV-1 isolate NL4-3; NEF_SIVMA: SIV isolate 1A11, as well as Nef clones representative of all major non-recombinant HIV-1 clades selected from the NIH HIV-1 sequence database as described elsewhere [45]. The first letter in each HIV-1 clone ID indicates the M-group subtype assignment (A1, A2, B, C, D, F1, F2, G, H, J and K). This figure was generated using Espript [46].

The Nef protein used in this study is derived from the HIV-1 B-clade isolate SF2, and consists of residues 51–205 (numbering is based on the crystal structure of Nef NL4-3 [22]). We chose Nef-SF2 for this project because it has been successfully crystallized in previous work [23] and has also been used in several drug discovery campaigns [11,12]. The crystal structure of Nef (51–205) was determined at 3.2 Å, with six Nef molecules forming three identical homodimers in the asymmetric unit. The overall fold of the Nef (51–205) core domain is quite similar to previous Nef structures [22,23,28] (**Fig 1A**), with a root-mean-square deviation (RMSD) between our new Nef structure and the search model (PDB ID: 1AVV) [28] of only 0.64 Å. The core domain consists of two anti-parallel α-helices (αA and αB), four central anti-parallel β-strands and a short C-terminal helix (αC; **Fig 2**). Electron density for the flexible central loop (150–176) was not observed, consistent with the disordered nature of this region in the absence of a binding partner. Notably, we observed one additional α-helix formed by residues 54–66 (designated helix α0), which is connected to the PXXP motif by residues 67-GFPVR-71 (**Fig 2**). The structure of this additional N-terminal helix was confirmed by simulated annealing, although most of the side chains are not completely resolved in the electron density map (**Fig 1B**). Helix α0 packs against helix αB and is connected to helix αA by a long loop containing the PXXP motif, so as to form an α0-αB-αA helix array (**Fig 1A**). Helix α0 contains the conserved acidic motif (62-EEEE-65), which interacts with the μ1 subunit of the AP-1 complex to downregulate MHC-I [26]. Since the acidic motif adopts an extended conformation in the context of the Nef/AP-1/MHC-I complex, helix α0 may represent a dynamic structural feature present only in the absence of AP-1 or other binding partners. The acidic motif is well-conserved across representative M-group HIV-1 Nef subtypes as well as SIV Nef, suggesting a common mechanism of downregulation of the MHC-I complex by primate immunodeficiency viruses (**Fig 2**).

### Nef (51–205) dimer interface and βOG binding pocket

**Fig 3:**
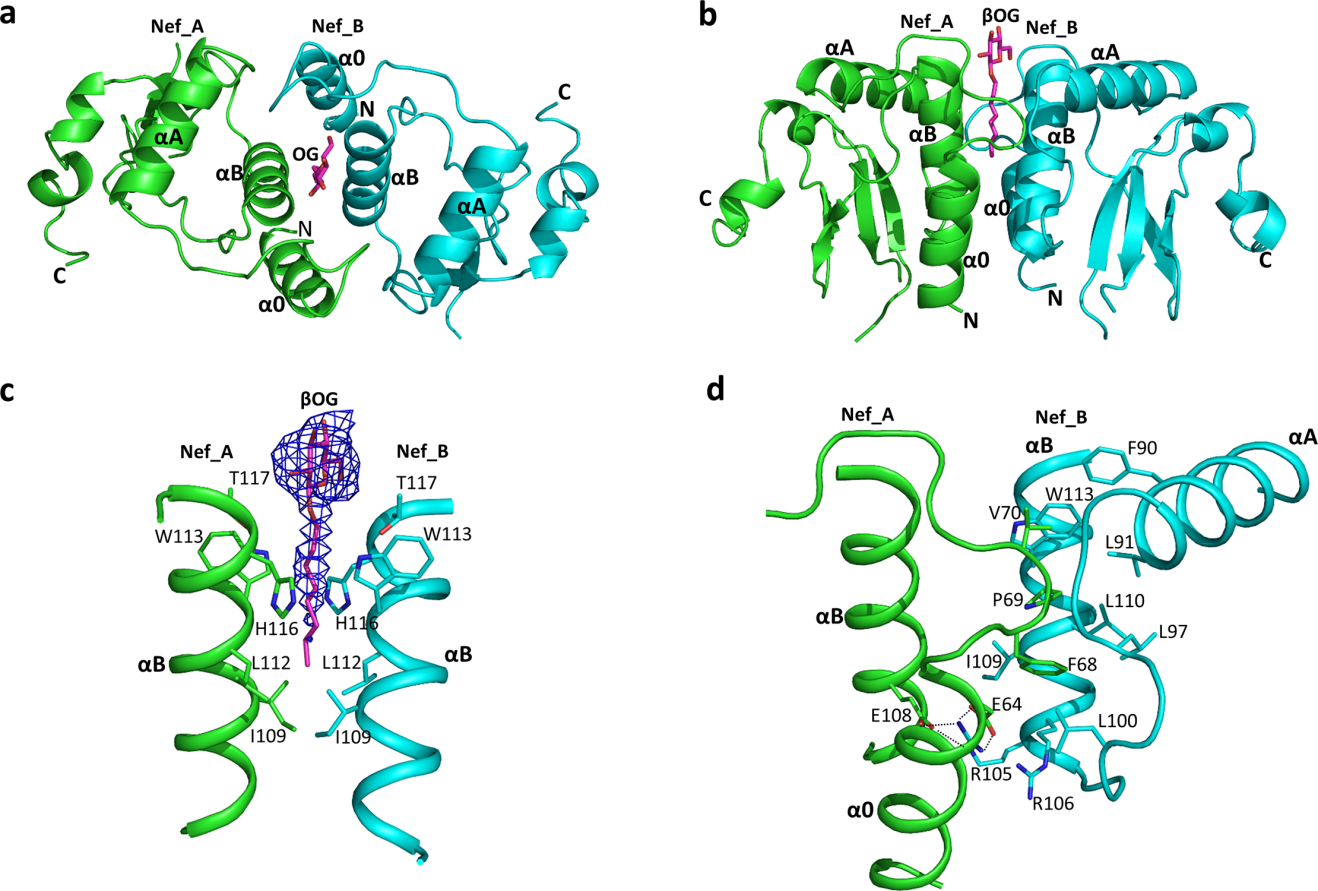
The overall structure of Nef (51–205) homodimer and residues forming the dimer interface. **(a)** The Nef (51–205) homodimer viewed from the top. The two Nef monomers are colored in green (Nef_A) and cyan (Nef_B), respectively, and helices α0, αA, and αB are indicated. The location of the βOG molecule is also shown (magenta sticks). **(b)** Side view of a Nef homodimer, with features labeled as in part a. **(c)** Close-up view of the contribution of helix B from each Nef monomer to the dimer interface; side chains of residues forming the hydrophobic pocket are indicated. The annealing omit map for βOG is colored in blue with a contour of 1.5 δ. **(d)** The interface between helix α0 and the PXXP loop from Nef_A and helices αA and αB from Nef_B; side chains from each Nef molecule involved in the interaction are shown in sticks.

Two Nef (51–205) molecules form a homodimer in the structure, and a representative dimer is shown in **Fig 3A**. The structures of the Nef monomers within the dimer are almost identical, with an RMSD of 0.36 Å, and are non-crystallographic symmetry (NCS) related molecules. The total buried surface area between the two Nef monomers is about 2000 Å^2^, with helices α0, αA and αB from each Nef monomer involved in dimerization (**Fig 3A and 3B**). Importantly, the dimer interface is not involved in crystal packing, consistent with potential physiological relevance.

As observed in previous Nef dimer structures [22,23,28], helix αB forms the core of the dimer interface (**Fig 3C**). Nef (51–205) helix B residues I109, L112, W113, H116, and the methyl group of T117 from both Nef monomers interact to create a hydrophobic pocket. The simulated annealed omit map revealed additional electron density in this pocket that was well-modeled by a single βOG molecule (**Fig 3C**). The hydrophobic tail of βOG inserts into the hydrophobic pocket formed by the side chains of I109, L112, W113 and H116 **(Fig 3B and 3C)**. All four residues are highly conserved across diverse M-group HIV-1 Nef subtypes, while residues I109, L112 and W113 are also conserved in SIV Nef (NEF_SIVMA, **Fig 2**). Unlike the hydrophobic tail, the polar head group of βOG is exposed to solvent and makes little contact with Nef. This observation raises the possibility that this Nef dimer may be induced or stabilized by interactions with other hydrophobic ligands through this hydrophobic pocket. The structure of the Nef (51–205) dimer interface observed here is quite different from other Nef dimer structures in which βOG was not used during purification [28,40]. The buried surface area of the Nef (51–205) dimer observed in the presence of βOG is also larger than that of previous dimeric Nef structures, suggesting that binding of a single βOG molecule stabilizes this unique dimer interface.

Consistent with prior Nef dimer structures, the conserved PXXP motif is not directly involved in the dimer interface, and is therefore free to interact with SH3 domains. However, the loop (67-GFPVR-71) connecting the PXXP motif with helix α0 from one Nef monomer (Nef-A, **Fig 3D**) closely contacts with the αA-loop-αB region from the other Nef molecule (Nef-B, **Fig 3D**). Residues F68, P69, and V70 in Nef-A are inserted into a groove between αA and αB from Nef-B and form hydrophobic contacts with F90, L91, L97, L100, R106, I109, L110, W113 and W183. E64 from Nef-A is positioned to form a salt bridge with R105 from Nef-B, while R105 also formed a hydrogen bond with E108 from helix αB of Nef-A (**Fig 3D**). Interestingly, Nef R105 also participates in the dimer interface of Nef crystal structures in complex with SH3 domains [22,28]. However, in this case, the αB helices realign so that R105 can form a salt bridge with D123. Most of the residues contributing to the dimer interface in the Nef (51–205) structure are well-conserved (**Fig 2**), suggesting that Nef proteins from multiple HIV-1 subtypes have the capacity to form a similar dimer in the absence of binding partners. Importantly, previous work has shown that mutagenesis of several of these residues, including I109, L112 and R105, disturbs Nef homodimerization in cells and also negatively impacts Nef functions related to enhancement of replication and CD4 downregulation [29].

### The Nef (51–205) dimer is distinct from other dimeric Nef structures

**Fig 4:**
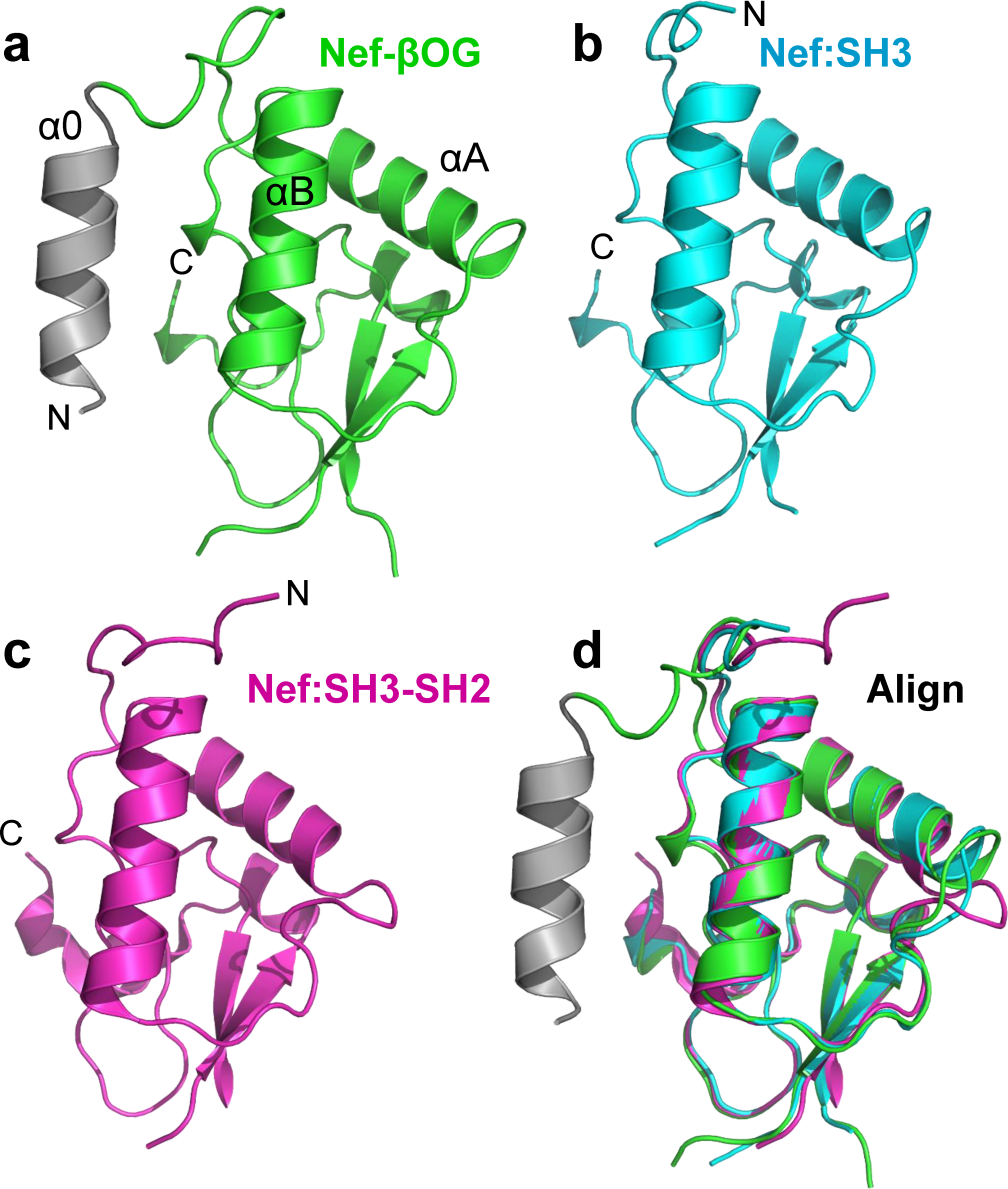
Conservation of the folded Nef core domain present in diverse dimer structures. Nef monomers shown are from **(a)** Nef (51–205)-βOG structure (green with N-terminal α0 helix shown in gray), **(b)** the Nef:SH3 complex (PDB: 1EFN), and **(c)** the Nef:SH3-SH2 complex (PDB: 4U5W). **(d)** Alignment of the core regions of one monomer from all three Nef complexes.

**Fig 5:**
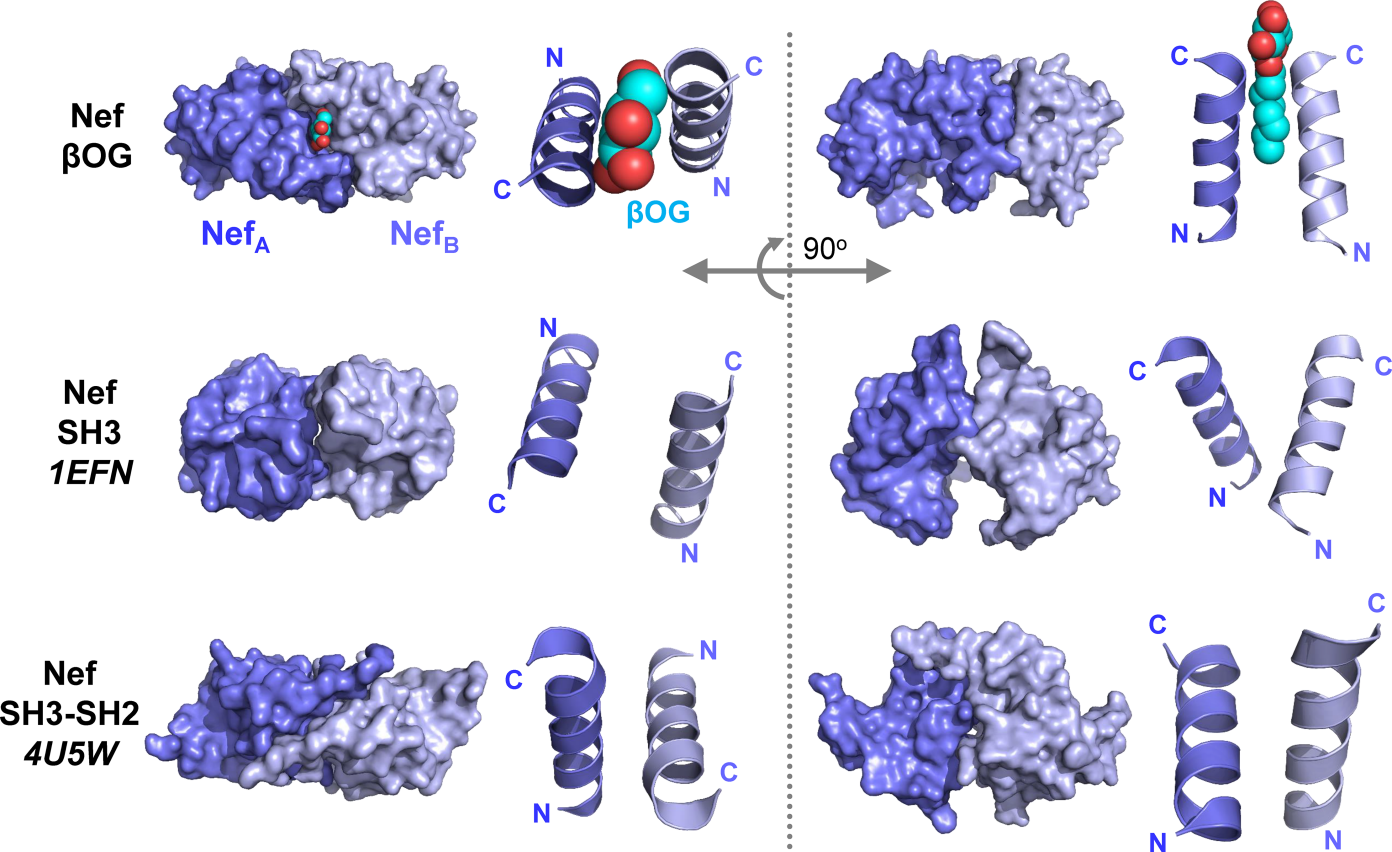
Comparison of dimer structures of Nef (51–205), Nef:SH3 and Nef:SH3-SH2. Models are presented for the crystal structures of Nef (51–205) with βOG (top row), the Nef dimer from the complex with the Fyn SH3 R96I mutant (PDB: 1EFN; middle row), and the Nef dimer from the complex with the Hck SH3-SH2 region (PDB: 4U5W; bottom row). The A and B monomers present in each Nef dimer are rendered in light and dark blue, respectively. The location of the βOG ligand is shown as a space-filling model in the Nef (51–205) structure. Two overall views of each dimer surface are shown, which are rotated along the X-axis by 90^○^ relative to one another. The relative positions of the αB helices which form the dimer interface are also shown next to each dimer.

Previous structural studies have shown that Nef forms dimers when bound to partner proteins derived from Src-family kinase regulatory domains. These include crystal structures of Nef in complex with the SH3 domain of Fyn (R96I mutant; PDB: 1EFN) as well as the dual SH3-SH2 regulatory unit of Hck (PDB: 4U5W). In both of these structures, as well as the Nef (51–205) structure presented here, the Nef core folds are remarkably similar with RMSDs of less than 1 Å (**Fig 4**). In these prior structures, the dimer interface is formed primarily by interactions of the αB helices. However, the Nef dimer interfaces present in these structures differ from one another, and as a result, the overall shape of each dimer and the relationship of the monomers to one another within each dimer are quite distinct. Two views of the overall surfaces of each dimer are modeled in **Fig 5**, along with the relative positions of the αB helices. In the Nef (51–205) dimer, the presence of βOG stabilizes a fairly compact and symmetrical dimer, with tight nearly parallel juxtaposition of the αB helices around the βOG molecule. In contrast, the αB helices are positioned almost at right angles to one another in the Nef-Fyn SH3-R96I structure. The αB helices in this dimer are also further apart, resulting in a less compact dimer overall. In the crystal complex with the Hck SH3-SH2 regulatory unit, the Nef dimer interface involves the αB helix and the loop connecting helices αA and αB [23]. Like Nef (51–205) in the presence of βOG, this dimer interface is also compact. However, the αB helices in this interface are orthogonally positioned relative to one another, resulting in an overall dimer structure distinct from the Nef:SH3 complex as well as Nef (51–205) with βOG.

Previous studies have shown that Nef dimerization is involved in the activation of Hck, as well as the recruitment of AP-2 as part of the CD4 downregulation pathway [30,41]. Recent hydrogen-deuterium exchange mass spectrometry analysis of Nef dynamics when complexed with the Hck SH3 domain vs. the SH3-SH2 dual domain clearly demonstrate that the helix αB forms the dimer interface in solution [42]. This analysis also showed that dimers resulting from Nef interaction with SH3 vs. SH3-SH2 are distinct, consistent with the crystal structures. Our finding that the presence of βOG can stabilize a third distinct dimer conformation illustrates the remarkably dynamic nature of Nef, despite structural conservation of the three-dimensional fold of the core region. If the unique Nef dimer that is stabilized by βOG binding interferes with Nef function, then the pocket that accommodates βOG may represent a previously unrecognized target for Nef inhibitor development. Recent studies have shown that small molecules predicted to bind within the dimer interface present in the Nef/SH3 crystal complex interfere with many Nef functions, including Src-family kinase activation, HIV-1 infectivity and replication and MHC-I and CD4 downregulation [12,18,30,43,44]. Our new structure suggests that drugs inducing a dimer structure similar to that observed with βOG may interfere with global Nef functions by preventing dynamic shifts in Nef conformation.

### β-octyl glucoside (βOG) induces Nef (51–205) dimer formation in solution

**Fig 6:**
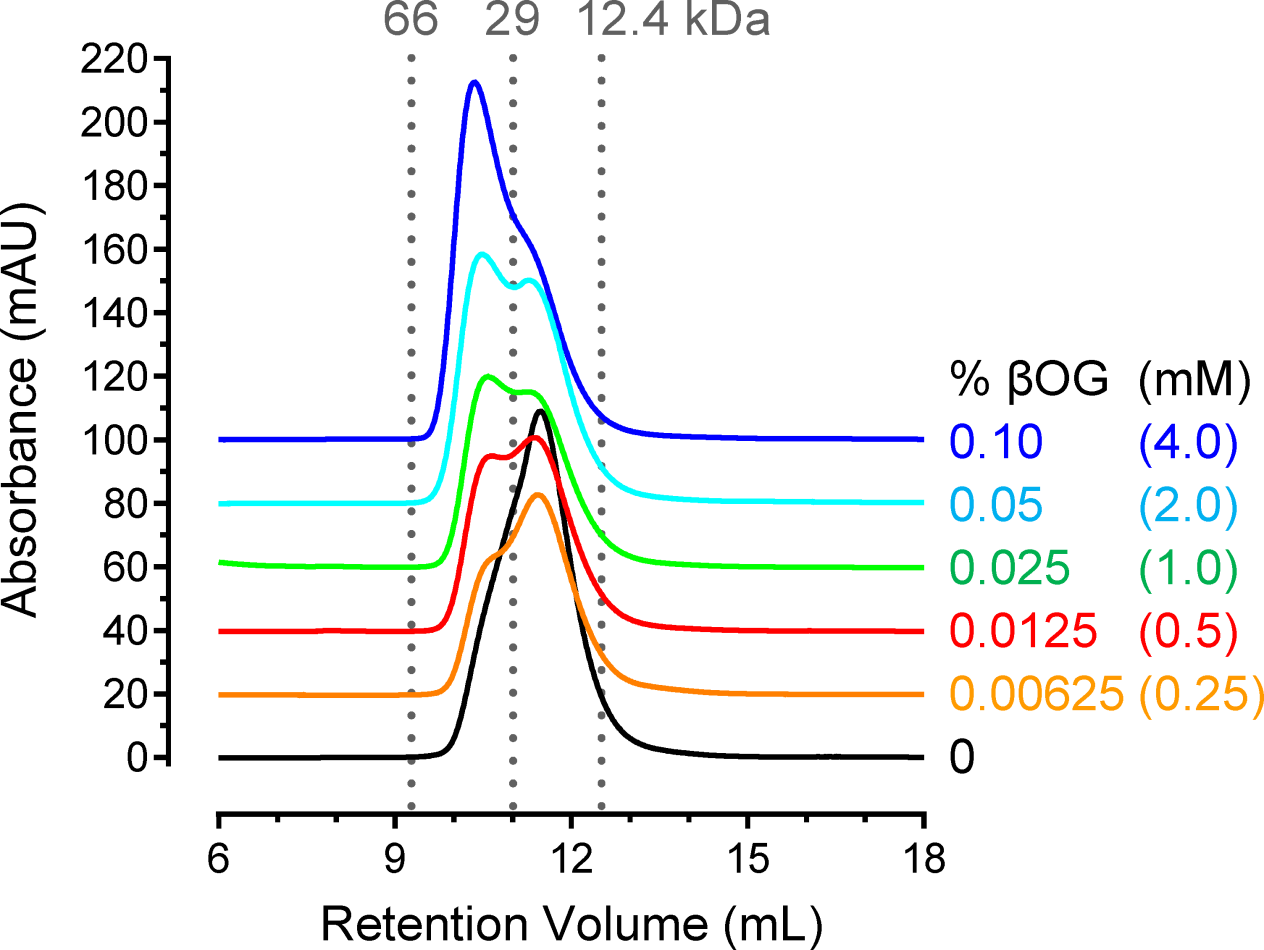
βOG induces Nef (51–205) dimer formation in solution. Recombinant purified Nef (51–205) was subjected to analytical size-exclusion chromatography on a Superdex 75 10/300 GL column (GE Healthcare Life Sciences) in the absence and presence of βOG over the range of concentrations shown. The concentration of Nef in the sample prior to loading was 549 μM in the absence and 494 μM in the presence of βOG. The elution profiles were determined by protein absorbance at 280 nm. Molecular weight standards are shown at the top, and include bovine serum albumin (66 kDa), carbonic anhydrase (29 kDa), and aprotinin (12.4 kDa). The Nef monomer eluted at a slightly lower retention volume than expected; this observation is most likely due to its long central flexible loop.

The crystal structure of Nef (51–205) presented above reveals a novel dimer interface with a unique hydrophobic pocket that accommodates the hydrophobic tail of a single molecule of βOG. Without βOG, Nef (51–205) did not crystallize under similar conditions, suggesting that the βOG molecule induces or stabilizes a unique Nef conformation essential for crystallization. To evaluate the effect of βOG on Nef dimerization in solution, we performed size exclusion chromatography in the presence and absence of low concentrations of this non-ionic surfactant. As shown in **Fig 6**, the recombinant Nef (51–205) protein eluted as a single and nearly symmetrical peak in the absence of βOG, consistent with a monomer. However, when the experiment was repeated in the presence of the same concentration of βOG used for the crystallization studies (0.1%), the Nef peak shifted to a lower retention volume, consistent with dimer formation. Subsequent two-fold dilutions of βOG revealed the presence of Nef monomer-dimer mixtures with as little as 0.00625% βOG, which corresponds to a detergent concentration of 250 μM against a Nef monomer protein concentration of just under 500 μM (**Fig 6**). Thus the detergent to Nef dimer stoichiometry in solution is approximately 1:1, consistent with the crystal structure. These results demonstrate that βOG induces Nef dimer formation in solution, an effect that is likely to occur through the hydrophobic pocket observed in the crystal structure.

## Conclusions

The structure of Nef (51–205) reported here represents a novel homodimer conformation that is induced by interaction with a single molecule of βOG. Many of the residues involved in the dimer interface and βOG-binding pocket are conserved across Nef alleles derived from multiple HIV-1 subtypes as well as SIV, suggesting that most Nef proteins have the potential to adopt the βOG -induced dimer structure. The finding that the dimer is stabilized by the hydrophobic tail of βOG both in the crystal and in solution raises the possibility that host cell lipids may also affect Nef dimerization, including the myristate modifications present on several Nef binding partners as well as Nef itself. Our results also highlight the remarkably dynamic capacity of HIV-1 Nef to form multiple quaternary structures, despite the highly conserved nature of its folded core region. Identification of small molecules that occupy the βOG-binding pocket reported here may restrict Nef dynamics and act as global inhibitors of the multiple functions of this critical HIV-1 virulence factor.
