# Correction: A single β-octyl glucoside molecule induces HIV-1 Nef dimer formation in the absence of partner protein binding

**DOI:** 10.1371/journal.pone.0196229

**Published:** 2018-04-17

**Authors:** The PLOS ONE Staff

The Funding statement is incorrect. The publisher apologizes for this error. The correct statement is: This work was supported by National Institute of Allergy and Infectious Diseases (grant nos. AI057083 and AI102724 to TES; contract no. HHSN272201400010I to Southern Research Institute). The funders had no role in study design, data collection and analysis, decision to publish, or preparation of the manuscript.
